# Usefulness of the Early Increase of Peripheral Blood Lymphocyte Count in Predicting Clinical Outcomes for Patients with Advanced Hepatocellular Carcinoma Treated with Durvalumab Plus Tremelimumab

**DOI:** 10.3390/cancers17081274

**Published:** 2025-04-09

**Authors:** Yuichi Honma, Michihiko Shibata, Masatoshi Ikemi, Kengo Yoshitomi, Nobuhiko Shinohara, Noriyoshi Ogino, Shinji Oe, Koichiro Miyagawa, Shintaro Abe, Masaru Harada

**Affiliations:** Third Department of Internal Medicine, School of Medicine, University of Occupational and Environmental Health, 1-1 Iseigaoka, Yahatanishi-ku, Kitakyushu 807-8555, Japan; 3shibata@med.uoeh-u.ac.jp (M.S.); m43174313@med.uoeh-u.ac.jp (M.I.); k.yoshitomi@med.uoeh-u.ac.jp (K.Y.); nbhkshnhr199112@gmail.com (N.S.); n-ogino@med.uoeh-u.ac.jp (N.O.); ooes@med.uoeh-u.ac.jp (S.O.); koichiro@med.uoeh-u.ac.jp (K.M.); s-abe@med.uoeh-u.ac.jp (S.A.); msrharada@med.uoeh-u.ac.jp (M.H.)

**Keywords:** durvalumab plus tremelimumab, hepatocellular carcinoma, initial response, neutrophil-to-lymphocyte ratio, peripheral lymphocyte, platelet-to-lymphocyte ratio

## Abstract

Background/Objectives: Durvalumab plus Tremelimumab (Dur/Tre) therapy is expected to have good therapeutic efficacy for patients with advanced hepatocellular carcinoma (HCC). However, the predictors of clinical response and prognosis have not been established. Here, we retrospectively investigated the predictors for therapeutic response and clinical prognosis of Dur/Tre therapy. Methods: This retrospective single-center study, which included 30 patients, aimed to evaluate predictors of treatment efficacy of Dur/Tre therapy for advanced HCC. Factors associated with an objective response rate (ORR), progression-free survival (PFS), overall survival (OS), and immune-mediated adverse events (imAEs) were examined. We especially focused on the initial change in peripheral lymphocyte count at 2 weeks after Dur/Tre introduction from baseline (Δlymphocyte). Results: Seventeen patients (56.7%) diagnosed with HCC BCLC stage C were enrolled. The median observational period was 11 months. The ORR and disease control rate (DCR) were 30.0% and 53.3%, respectively. The median PFS was 3.7 months and OS was not reached. The high Δlymphocyte was an independent predictor of objective response (hazard ratio [HR], 1.004; *p* = 0.016). The high Δlymphocyte (above +245/µL) was an independent predictive factor for better PFS (HR, 0.308; 95% CI, 0.095–0.998; *p* = 0.049), and the median PFS was significantly prolonged in the high Δlymphocyte (above +245/µL) compared to low Δlymphocyte (less than +245/µL) (not reached vs. 1.96 months, log-rank *p* = 0.003). Conclusions: In Dur/Tre therapy, the early increase in peripheral blood lymphocyte count was useful for predicting clinical response and prognosis.

## Simple Summary

There are no standard biomarkers in advanced hepatocellular carcinoma (HCC) which effectively predict response or resistance to durvalumab plus tremelimumab. We investigated the correlation between peripheral lymphocyte count and clinical outcomes in patients with advanced HCC treated with durvalumab plus tremelimumab. Our results demonstrated that the initial change in peripheral lymphocyte count at 2 weeks after durvalumab plus tremelimumab introduction from the baseline (Δlymphocyte) was significantly correlated to objective responses. Furthermore, the high Δlymphocyte (above +245/µL) was an independent predictive factor for better progression-free survival (PFS), and the median PFS was significantly prolonged in the high Δlymphocyte (above +245/µL) compared to low Δlymphocyte (less than +245/µL). Thus, an early peripheral blood lymphocyte response, such as the increase in peripheral blood lymphocyte count at 2 weeks after the introduction of a single priming dose of tremelimumab, may be a biomarker and useful for predicting objective response and better PFS in advanced HCC treated with durvalumab plus tremelimumab.

## 1. Introduction

Hepatocellular carcinoma (HCC) is one of the most commonly diagnosed malignancies and the sixth leading cause of cancer-related death worldwide [1]. Systemic chemotherapy regimens including molecular targeted agents (MTAs) and immune checkpoint inhibitors (ICIs) have become the standard treatment option in a subset of patients with unresectable or advanced HCC [2]. Systemic chemotherapy with MTAs was first approved with sorafenib, which was characterized by a low objective response rate (ORR) and a prominent disease control rate (DCR) [3]. Regorafenib was approved as a second-line treatment which prolongs the overall survival (OS) sequential administration in patients with progressive sorafenib [4,5]. Lenvatinib was next approved as a first-line treatment which achieved a non-inferior OS and a superior ORR and progression-free survival (PFS) to that of sorafenib [6,7]. Following these MTAs, ramucirumab [8] and cabozantinib [9] were approved as second-line treatment. ICIs and their combination can induce robust and durable clinical response. The IMbrave150 trial demonstrated that the combination of atezolizumab, programmed cell death ligand-1 (PD-L1) inhibitor, and bevacizumab, vascular endothelial growth factor (VEGF) inhibitor, improved OS compared to sorafenib [10,11]. In addition, HIMALAYA trial showed that the combination of durvalumab, PD-L1 inhibitor, and tremelimumab, cytotoxic T lymphocyte-associated antigen 4 (CTLA-4) inhibitor, also improved OS compared to sorafenib [12,13].

To date, there are no standard biomarkers in advanced HCC which effectively predict response or resistance to ICI therapies. Thus, the discovery of potentially predictive and prognostic biomarkers for the efficacy of ICI therapies in patients with HCC has gained interest. While PD-L1 expression is a validated predictive biomarker of ICI response in some other solid malignancies [14], it has not been consistently correlated with response to ICI therapies in HCC. Previous studies have demonstrated the usefulness of pretreatment neutrophil-to-lymphocyte ratio (NLR), one of the most widely studied inflammatory markers, as a predictive marker for clinical outcome in patients with HCC treated with atezolizumab plus bevacizumab (Atez/Bev) [15,16,17]. Furthermore, in addition to baseline NLR, the NLR at the start of the second course of Atez/Bev was also significantly associated with clinical response and prognosis in Atez/Bev therapy [18].

One of the most notable features of durvalumab plus tremelimumab (Dur/Tre) is a single high priming dose of tremelimumab, termed STRIDE (Single Tremelimumab Regular Interval Durvalumab) regimen. The number of peripheral lymphocytes, associated with Ki67+ subset of CD8+ T cells, has been reportedly elevated on day 15 and is associated with the objective response in patients who received STRIDE regimen [19]. Therefore, we hypothesized that the peripheral lymphocytes during Dur/Tre therapy may alter and can more clearly reflect clinical outcomes than Atez/Bev therapy.

In this study, we investigated the change in peripheral lymphocyte count between at initiation of Dur/Tre and 2 weeks after Dur/Tre introduction, and the correlation between initial changes in the peripheral lymphocyte count and clinical outcomes in patients with advanced HCC treated with Dur/Tre therapy.

## 2. Materials and Methods

### 2.1. Patients

In this single-center, retrospective study, the data of patients with advanced HCC who received Dur/Tre treatment at our hospital from March 2023 to October 2024 were collected. We diagnosed patients with HCC by radiological criteria of contrast-enhanced computed tomography (CT) or Gd-EOB-DTPA magnetic resonance imaging (MRI) and/or liver biopsy. We defined the macrovascular invasion as the presence of a portal vein tumor thrombus, hepatic vein tumor thrombus, and/or bile duct invasion of HCC. A total of 32 patients who received Dur/Tre, including 12 patients who received Atez/Bev prior to Dur/Tre, were examined in the present study. We excluded 2 patients treated with an observation period of less than 30 days and/or without evaluating the therapeutic effect by contrast-enhanced CT or Gd-EOB-DTPA MRI. Finally, we analyzed 30 patients with unresectable HCC. The date when Dur/Tre started was defined as the start of the follow-up. The end of the follow-up was defined as the date of the final visit for patients who remained alive and the date of death for patients who died during follow-up. This study was censored on 28 October 2024.

### 2.2. Treatment Protocol and Evaluation of Therapeutic Response

The decision to introduce Dur/Tre treatment was made by attending a physician and based on the Japan Society of Hepatology algorithm [20]. Dur/Tre treatment protocol consisted of 1500 mg durvalumab plus 300 mg tremelimumab on day 1 and subsequently 1500 mg durvalumab every 4 weeks according to the manufacturer’s guideline. The initial therapeutic response was evaluated using contrast-enhanced CT and/or Gd-EOB-DTPA MRI at approximately 4–8 weeks after the introduction of Dur/Tre treatment according to the tumor markers or the patient’s condition. The therapeutic response was evaluated in accordance with the Response Evaluation Criteria in Solid Tumors (RECISTs) version 1.1 [21]. A complete response (CR) was defined as the disappearance of all lesions. A partial response (PR) was defined as 30% or greater decrease in the sum of diameters of target lesions, taking as reference the baseline sum of diameters of the target lesions. A progressive disease (PD) was defined as 20% or greater increase in the sum of diameters of the target lesions, taking as reference the smallest sum in the study (this includes the baseline sum if that is the smallest). In addition to the relative increase of 20%, the sum must also demonstrate an absolute increase of at least 5 mm. The appearance of 1 or more new lesions is also considered progression. A stable disease (SD) was defined as neither sufficient shrinkage to qualify for PR nor sufficient increase to qualify for PD. ORR was defined as the sum of the CR and PR rates and DCR was defined as the sum of CR, PR, and SD. PFS was defined as the time from the date of Dur/Tre therapy initiation to the date of documented PD per RECIST version 1.1 or death (regardless of the cause of death). If the patient was lost to follow-up before PD, the PFS was censored at the date of the last observation. OS was calculated from the date of Dur/Tre therapy initiation to the date of death or the last follow-up.

### 2.3. Laboratory Tests

Hematologic and blood chemistry tests were carried out using standard assays. The NLR, lymphocyte-to-monocyte ratio (LMR), and platelet-to-lymphocyte ratio (PLR) were calculated from the absolute neutrophil, monocyte, lymphocyte, and platelet counts, measured in the peripheral blood. The albumin–bilirubin (ALBI) score was calculated based on serum albumin and total bilirubin values, using the following formula: ALBI score = (log_10_ bilirubin [μmol/L] × 0.66) + (albumin [g/L] × −0.085) [22].

### 2.4. Assessment of Adverse Events

Adverse events were assessed using the National Cancer Institute Common Terminology Criteria for Adverse Events version 5.0 (https://ctep.cancer.gov/protocoldevelopment/electronic_applications/ctc.htm [accessed on 31 October 2024]). Immune-mediated adverse events (imAEs) were diagnosed by the attending physician. Administration was discontinued in patients with unacceptable serious imAEs or clinical progression of the tumors. After discontinuing Dur/Tre treatment, the attending physicians made decisions on the introduction of another treatment.

### 2.5. Statistical Analysis

We presented continuous variables as median and interquartile (IQR). ORR and DCR were compared using the chi-square test and Fisher’s exact test. Correlation coefficients were determined by Spearman’s rank correlation coefficient analysis. For evaluations of PFS and OS following the introduction of Dur/Tre, the Kaplan–Meier method and a log-rank test were used. Prognostic factors related to ORR were analyzed by logistic regression analysis and PFS and OS were analyzed by Cox proportional hazard analysis, respectively. Factors with a *p* value less than 0.05 in univariate analysis were used for multivariate analysis.

The prognostic factors analyzed in the present study were age, gender, treatment line (first-line or later-line), presence or absence of imAEs, etiology of liver disease (viral or non-viral), alpha-fetoprotein (AFP, above or less than 400 ng/mL), peripheral white blood cell (WBC) count, lymphocyte count, monocyte count, neutrophil count, NLR, LMR, PLR at the time of Dur/Tre introduction. We also examined NLR, LMR, and PLR at 2 weeks after Dur/Tre introduction (NLR2w, LMR2w, and PLR2w, respectively), and the degree of change in the amount of neutrophil, monocyte and lymphocyte from at Dur/Tre introduction to at 2 weeks after Dur/Tre introduction (Δneutrophil, Δmonocyte, and Δlymphocyte, respectively). A receiver operating characteristic (ROC) curve analysis was performed to determine the cutoff values for predicting an objective response in logistic regression analysis. *p* values less than 0.05 were considered statistically significant. All statistical analyses were performed using the Statistical Package for the Social Sciences Software Program (SPSS) version 25.0 (IBM, Tokyo, Japan).

## 3. Results

### 3.1. Patient Characteristics

The characteristics of the patients at Dur/Tre introduction and at 2 weeks after Dur/Tre introduction are shown in Table 1. The median age was 75 years and 24 patients (80.0%) were male. Dur/Tre was introduced as the first-line in 15 patients (50.0%) and as the second- or later-line in 15 patients (50.0%). The etiologies of HCC were viral hepatitis (hepatitis B in 8 patients and hepatitis C in 6 patients) in 14 patients (46.7%), non-viral hepatitis (alcohol-associated liver disease [ALD] in 9 patients and metabolic dysfunction-associated steatotic liver disease [MASLD] in 7 patients) in 16 patients (53.3%), and cirrhosis in 19 patients (63.3%). The etiologies of cirrhosis were hepatitis B in six patients (31.6%), hepatitis C in three patients (15.8%), ALD in six patients (31.6%), and MASLD in four patients (21.0%). Child–Pugh class was A in 27 patients (90.0%) and modified ALBI grade was 1 in 6 (20.0%). A total of 17 patients (56.7%) had Barcelona Clinic Liver Cancer (BCLC) stage C. The median maximum tumor size was 34 mm and the tumors exceeded the up-to-seven criteria, defined based on the sum of the maximum tumor diameter (cm) and the number of tumors in the liver [23], in all patients. A total of 8 patients (26.7%) had macrovascular invasion, and 17 patients (56.7%) had extrahepatic spread. The median peripheral WBC count was 5750/µL, lymphocyte count was 1287/µL, monocyte count was 351/µL, and neutrophil count was 3763/µL, respectively. The median values of NLR2w, LMR2w, and PLR2w were 3.05 (IQR, 2.06–4.13), 3.16 (IQR, 2.13–4.03), and 119.9 (IQR, 72.4–163.8), respectively. The median values of Δneutrophil, Δmonocyte, and Δlymphocyte were +286.9/µL (IQR, −419.1–1225.2), +57.3/µL (IQR, −10.3–153.1), and +35.3/µL (IQR, −132.3–369.8), respectively. The median observation period was 11.07 months (IQR, 4.85–15.75).

### 3.2. Therapeutic Efficacy

**Figure 1:**
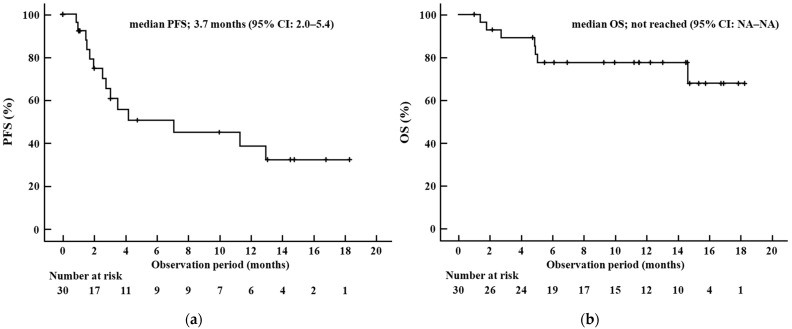
Kaplan–Meier curves for (**a**) progression-free survival and (**b**) overall survival in the whole cohort. Abbreviations: PFS, progression-free survival; OS, overall survival; CI, confidence interval; NA, not available.

During the observation period, six patients continued Dur/Tre. A total of 1 patient discontinued Dur/Tre after achieving CR after conversion surgery, 1 patient discontinued as liver failure due to HCC progression, 13 patients discontinued as PD, and 9 patients discontinued as AEs. The median PFS in the whole cohort was 3.7 months (95% confidence interval [CI], 2.0–5.4) (Figure 1a). The median OS in the whole cohort was not reached and the 95% CI for OS could not be calculated (Figure 1b).

The clinical response of the entire cohort is demonstrated in Table S1. The radiological best response rates for CR, PR, SD, and PD were 6.7%, 23.3%, 23.3%, and 46.7%, respectively. One patient with multinodular HCC discontinued Dur/Tre as CR by conversion surgery following achieved shrinkage and disappearance of HCCs during Dur/Tre therapy. Because the histopathological findings revealed no viable HCC lesion in the whole liver resection specimen, this case was considered to achieve pathological CR by treating with Dur/Tre alone. But, except for this patient, no other patient received conversion surgery or other locoregional therapy, and other systemic therapy before PD during Dur/Tre. ORR and DCR were 30.0% and 53.3%, respectively. There were no significant differences in ORR and DCR between first-line and later-line Dur/Tre treatment in the present cohort.

### 3.3. Analysis of Prognostic Factors for Objective Response

We performed univariate and multivariate logistic regression analysis to determine the predictors associated with objective response (Table 2). Univariate analysis demonstrated that PLR2w (hazard ratio [HR], 0.973; 95% CI, 0.951–0.995; *p* = 0.017) and Δlymphocyte (HR, 1.004; 95% CI, 1.001–1.006; *p* = 0.016) as the statistically significant factors correlated with an objective response. But these were not statistically significant based on multivariate analysis. We also investigated the correlation between PLR2w and Δlymphocyte. PLR2w and Δlymphocyte showed a significantly negative correlation (r = −0.538, *p* = 0.002) (Figure S1).

We constructed an ROC curve to identify an accurate threshold for the use of PLR2w and Δlymphocyte in predicting objective response. Both PLR2w (area under the curve [AUC], 0.831; 95% CI, 0.627–1.000; *p* = 0.005) and Δlymphocyte (AUC, 0.761; 95% CI, 0.571–0.951; *p* = 0.031) were closely associated with objective response. According to the ROC analysis, the optimal cut-off value of PLR2w and Δlymphocyte were 98.6 and +244.5/µL, respectively. Thus, we divided into two groups by the PLR2w value of 98.6 and by the Δlymphocyte value of +245/µL.

The ORR was significantly higher in the low PLR2w (less than 98.6) group (*n* = 10) than the high PLR2w (above 98.6) group (*n* = 20) (80.0% vs. 5.0%; *p* < 0.001). It was significantly higher in the high Δlymphocyte (above +245/µL) group (*n* = 10) than the low Δlymphocyte (less than +245/µL) group (*n* = 20) (70.0% vs. 10.0%; *p* = 0.002). The DCR was also significantly higher in the low PLR2w group than the high PLR2w group (90.0% vs. 35.0%; *p* = 0.006). It was significantly higher in the high Δlymphocyte group than the low Δlymphocyte group (90.0% vs. 35.0%; *p* = 0.006) (Table 3).

We also divided into two groups the “minus data” and “plus data” of Δlymphocyte. The ORR was significantly higher in the plus Δlymphocyte group (*n* = 17) than the minus Δlymphocyte group (*n* = 13) (47.0% vs. 7.6%; *p* < 0.001).

### 3.4. Analysis of Prognostic Factors for PFS

We performed univariate and multivariate Cox regression analyses for PFS. Peripheral WBC count, lymphocyte count, monocyte count, neutrophil count, NLR, LMR, and PLR at the time of Dur/Tre introduction were not associated with the PFS. Univariate Cox regression analyses revealed that presence of imAEs (HR, 0.239; 95% CI, 0.086–0.667; *p* = 0.006), NLR2w (HR, 1.129; 95% CI, 1.003–1.271; *p* = 0.044), PLR2w (HR, 1.008; 95% CI, 1.001–1.016; *p* = 0.023), and the high Δlymphocyte (above +245/µL) (HR, 0.215; 95% CI, 0.072–0.647; *p* = 0.006) were statistically significant predictive factors for better PFS. Multivariate Cox regression analyses revealed that presence of imAE (HR, 0.321; 95% CI, 0.112–0.923; *p* = 0.035) and the high Δlymphocyte (HR, 0.308; 95% CI, 0.095–0.998; *p* = 0.049) were independent predictive factors for better PFS (Table 4).

**Figure 2:**
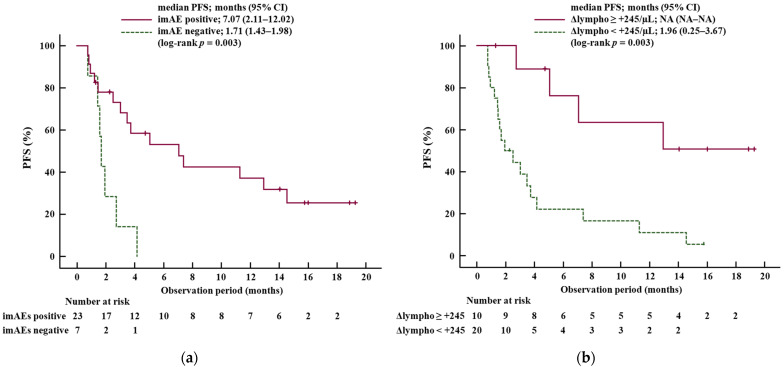
Kaplan–Meier curves for progression-free survival (PFS). (**a**) The median PFS in patients with immune-mediated adverse events (imAEs) (imAE positive, solid line) was significantly better than that in patients without imAEs (imAE negative, dotted line). (**b**) The median PFS stratified by the optimal value of Δlymphocyte (+245/µL) at 2 weeks after durvalumab plus tremelimumab introduction. The median PFS in the high Δlymphocyte group (solid line) was significantly better than that in the low Δlymphocyte group (dotted line). Abbreviations: PFS, progression-free survival; imAEs, immune-mediated adverse events; Δlymphocyte, the change in the amount of lymphocyte count between at baseline and at 2 weeks after durvalumab plus tremelimumab introduction; CI, confidence interval; NA, not available.

The median PFS time was 7.07 months (95% CI, 2.11–12.02) in patients with imAEs and 1.71 months (95% CI, 1.43–1.98) in patients without imAEs (*p* = 0.003) (Figure 2a). The median PFS time was not reached in the high Δlymphocyte group and 1.96 months (95% CI, 0.25–3.67) in the low Δlymphocyte group (*p* = 0.003) (Figure 2b).

The median PFS time was 7.07 months (95% CI, 0.00–18.20) in the plus Δlymphocyte group and 1.96 months (95% CI, 0.94–2.98) in the minus Δlymphocyte group (*p* = 0.032) (Figure S2).

We further analyzed 29 patients excluding one case with conversion surgery. The median PFS time was 12.92 months (95% CI, 0.00–27.72) in the high Δlymphocyte group and 1.96 months (95% CI, 0.25–3.67) in the low Δlymphocyte group (*p* = 0.008) (Figure S3).

### 3.5. Analysis of Prognostic Factors for OS

Univariate Cox regression analyses revealed that imAEs presence (HR, 0.129; 95% CI, 0.028–0.600; *p* = 0.009) and AFP above 400 ng/mL (HR, 4.829; 95% CI, 1.143–20.409; *p* = 0.032) were statistically significant predictive factors for better OS. Multivariate Cox regression analyses revealed that both imAEs presence (HR, 0.111; 95% CI, 0.019–0.662; *p* = 0.016) and AFP above 400 ng/mL (HR, 10.848; 95% CI, 1.804–65.229; *p* = 0.009) were statistically significant predictive factors for OS (Table S2).

The median OS time was not reached in patients with imAEs and 4.89 months (95% CI, 2.27–7.50) in patients without imAEs (*p* = 0.002) (Figure S4a). The median OS time was 12.32 months (95% CI, 0.00–26.84) in patients with AFP ≥ 400 ng/mL and not reached in patients with AFP < 400 ng/mL (*p* = 0.018) (Figure S4b).

### 3.6. Analysis of Predictive Factors for imAEs Due to Dur/Tre Therapy

The imAEs in the present whole cohort are demonstrated in Table S3. At the time of analysis, the most common grade 3 or higher imAEs were colitis/diarrhea (*n* = 5) following hypopituitarism (*n* = 3), interstitial pneumonia (*n* = 2), and hepatitis (*n* = 2). Univariate logistic regression analyses demonstrated that peripheral WBC count, neutrophil count, monocyte count, and NLR at the time of Dur/Tre introduction were statistically significant predictive factors for the occurrence of any grade imAEs. However, these were not statistically significant based on multivariate analysis (Table S4). In addition, there was no statistically significant predictive factors for grade 3 or higher imAEs based on univariate analyses.

## 4. Discussion

To date, various systemic chemotherapeutic treatments have been approved for advanced HCC. In the STRIDE regimen, the ORR reported 20.1% with a median time to response of 2.17 months in the HIMALAYA study [12]. This anti-CTLA-4 antibody containing regimen showed a clear tail plateau in the Kaplan–Meier curve. Among the long-term survivors treated with STRIDE regimen, above half of the participants had an objective response by RECIST version 1.1 [13]. The median PFS was 3.78 months in the HIMALAYA study [12]. The median PFS in the real-world clinical practice was previously demonstrated as 3.0 to 3.9 months in large cohort studies [24,25]. Thus, the median PFS in the present study seems to be comparable with those in previous reports. Extending PFS is considered to lead to extended OS in ICI therapy for advanced HCC [26]. Thus, the achievement of the objective response, especially in the early phase after Dur/Tre initiation, seems to be important for extending OS in Dur/Tre therapy. Early changes in tumor markers, AFP and des-gamma-carboxy prothrombin (DCP), after Dur/Tre introduction have been reported to be a useful biomarker for predicting objective response and clinical prognosis in Dur/Tre therapy [27,28].

An understanding of the immune responses to cancer should include the role of the peripheral immune system as well as the tumor microenvironment [29]. Thus, we focused on a simple tool “peripheral blood lymphocyte count”. Although peripheral lymphocytes have the advantages of low invasiveness and real-time efficacy monitoring, the relationship between peripheral lymphocytes and clinical outcomes in HCC seems to be very complex and remains unclear. Therefore, the value of peripheral lymphocytes and their subsets in predicting the efficacy of ICI therapy in patients with advanced HCC needs further investigation. The NLR, recognized as an effective indicator of systemic inflammatory response, has been reported to correlate with the efficacy and prognosis of immunotherapy in HCC [30,31]. The higher NLR has been recognized as a predictive factor for poor prognosis in patients with advanced HCC. Neutrophils have been recognized to be a main source of circulating VEGF, neutrophilia could contribute to tumor angiogenesis and metastases by providing an appropriate microenvironment for HCC progression through the release of VEGF [32]. VEGF also exhibits immunosuppressive functions to generate immunosuppressive tumor microenvironment and promote cancer immune escape [33]. On the other hand, activated and proliferating lymphocytes are thought to be promoting patient survival through mechanisms leading to the inhibition of tumor survival and proliferation [34].

The low NLR (NLR ≤ 3) at Dur/Tre introduction was reported to be significantly associated with better PFS [28]. While, the value of NLR as well as the count of neutrophil and lymphocyte and the value of LMR and PLR at Dur/Tre initiation were not correlated with objective response and PFS in our study. Thus, we hypothesized that the value of NLR and the count of neutrophil and lymphocyte at 2 weeks after Dur/Tre therapy introduction can better reflect a patient’s immunological status, according to the previous clinical trial [19]. Notably, our results demonstrated that PLR2w and Δlymphocyte correlated with the objective response with statistical significance. On the other hand, NLR2w and Δneutrophil were not recognized as a predictive factor for objective response. The ability of the immune response in ICI therapy has been known to decrease with age [35]. Most of the patients (86.7%) in this cohort were over the age of 65 years in the present study. We did not recognize the correlation between age and Δlymphocyte.

We next divided patients into two groups by the optimal cut-off value of PLR2w (98.6) and Δlymphocyte (+244.5/µL) according to the ROC analysis. Interestingly, the low PLR2w group and the high Δlymphocyte group demonstrated significantly high ORR and DCR. Furthermore, the high Δlymphocyte (above +245/µL) was demonstrated as the independent predictor for better PFS with statistical significance (*p* = 0.049). Although the observation period might not have been long enough in this study, PFS was clearly stratified comparing the high Δlymphocyte group and the low Δlymphocyte group. The high Δlymphocyte group exhibited a significantly better PFS (*p* = 0.003). Univariate Cox regression analysis showed that the value of NLR2w and PLR2w as significant predictors for better PFS, but these were not statistically significant in multivariate Cox regression analysis. Because PLR2w and Δlymphocyte were negatively correlated in this study, the low value of PLR2w seems to be due to an increase in the lymphocyte count at 2 weeks after Dur/Tre introduction. Therefore, we considered the high Δlymphocyte (above +245/µL) as the most valuable predictive factor for better PFS.

The baseline AFP value, above 400 ng/mL, was an independent predictor of poor OS. The occurrence of imAEs has been reported to correlate the efficacy and prognosis of Atez/Bev therapy in advanced HCC [36]. Our results demonstrated that the presence of imAEs was an independent predictive factor for better PFS and OS in Dur/Tre therapy. Currently, there are no prospectively validated biomarkers to predict the onset of severe imAEs in Dur/Tre therapy. We analyzed the contributing factors to the occurrence of imAEs. Multivariate analysis demonstrated no significant predictors for imAEs. Although peripheral lymphocyte count is not associated with occurrence of imAEs, this hypothesis requires further exploratory validation.

Immunohistochemistry-based examination of tumor biopsy specimens has been reported to be useful for identifying subgroups showing good response to ICI in advanced HCC [37]. The usefulness of immunohistochemistry of CD8+ tumor-infiltrating lymphocytes of the tumor biopsy specimen has also been reported for predicting clinical response in Dur/Tre therapy [38]. However, it seems to be difficult to perform tumor biopsy in all patients with advanced HCC. Several patients have tumor markers within the normal limit at Dur/Tre introduction. Thus, tumor markers may not reflect the clinical efficacy and prognosis of all patients treated with Dur/Tre. On the other hand, the value of Δlymphocyte is easily obtained in routine medical care, so it is considered to be a non-invasive and cost-effective tool. However, this study has several limitations as well. First, this study was a retrospective analysis based on a single center and included some potential biases, such as treatment selection. Second, the sample size was very small and the observation period was relatively short. Finally, we did not analyze the immune cell profiles, including flow cytometry for analyzing lymphocyte subsets. A previous report demonstrated a significant increase in the percentage of CD3+CD8+ T cells after ICI therapy compared with baseline in patients with HCC [39]. Moreover, the peripheral Ki67+ subset of CD8+ T cells has been reported to increase early during STRIDE regimen, and which was associated with objective response [19]. Thus, the increase in the peripheral lymphocytes early during Dur/Tre therapy in the present study may reflect the increase in peripheral CD8+ T cells. This study is non-prospective design analysis and cannot confirm the definitive conclusions regarding the predictive value of Δlymphocyte. However, our study could be considered hypothesis-generating and warrants further validation study in a large cohort for a longer observation period.

## 5. Conclusions

An early increase in the peripheral blood lymphocyte count at 2 weeks after the introduction of a single priming dose of tremelimumab may be a biomarker and useful for predicting objective response and better PFS in patients with advanced HCC treated with STRIDE regimen.

## Figures and Tables

**Table 1:** Characteristics of the patients at durvalumab plus tremelimumab introduction and at 2 weeks after durvalumab plus tremelimumab introduction.

Factor	Data (*n* = 30)
Age, years	75 (69, 77)
Gender, male/female, *n*	24/6
ECOG PS, 0/1, *n*	26/4
Dur/Tre treatment line, 1st/later, *n*	15/15
Etiology, hepatitis B/hepatitis C/non-viral, *n*	8/6/16
Liver cirrhosis, yes/no, *n*	19/11
Child–Pugh class, A/B, *n*	27/3
Modified ALBI grade, 1/2a/2b/3, *n*	6/8/14/2
Max size of tumor, mm	34 (22, 72)
Macrovascular invasion, positive/negative, *n*	8/22
Extrahepatic spread, positive/negative, *n*	13/17
BCLC stage, A/B/C, *n*	1/12/17
History of Atez/Bev, yes/no, *n*	12/18
Laboratory data at Dur/Tre introduction	
Albumin, g/dL	3.5 (3.2–3.8)
Total bilirubin, mg/dL	0.9 (0.6–1.2)
AFP, ng/mL	16.7 (5.2–7929.4)
DCP, mAU/mL	636.0 (55.5–3864.7)
Platelet count, ×10^4^/μL	15.7 (11.3–19.2)
WBC count, /μL	5750 (4275–7050)
Neutrophil count, /μL	3763 (2502–5131)
Monocyte count, /μL	351 (260–469)
Lymphocyte count, /μL	1287 (1052–1582)
NLR	3.27 (1.96–4.47)
LMR	3.57 (2.80–4.79)
PLR	122.9 (91.3–167.6)
Laboratory data at 2 weeks after Dur/Tre introduction	
Platelet count, ×10^4^/μL	14.5 (10.8–21.0)
WBC count, /μL	6350 (5000–8925)
Neutrophil count, /μL	4187 (2648–5560)
Monocyte count, /μL	468 (297–646)
Lymphocyte count, /μL	1360 (1052–1786)
NLR	3.05 (2.06–4.13)
LMR	3.16 (2.13–4.03)
PLR	119.9 (72.4–163.8)
Observational period, months	11.07 (4.85–15.75)

Data displayed as median (interquartile range). Abbreviations: Dur/Tre, durvalumab plus tremelimumab; ECOG PS, Eastern Cooperative Oncology Group performance status; ALBI, albumin–bilirubin; BCLC, Barcelona Clinic Liver Cancer; Atez/Bev, atezolizumab plus bevacizumab; AFP, alpha-fetoprotein; DCP, des-γ-carboxy prothrombin; WBC white blood cell; NLR, neutrophil-to-lymphocyte ratio; LMR, lymphocyte-to-monocyte ratio; PLR, platelet-to-lymphocyte ratio.

**Table 2:** Logistic regression analysis to determine the predictors associated with objective response.

		Univariate Analysis			Multivariate Analysis		
		HR	(95% CI)	*p* Value	HR	(95% CI)	*p* Value
Age	years	1.002	(0.928–1.083)	0.953			
Gender	male/female	0.333	(0.053–2.115)	0.244			
Etiology	viral/non-viral	0.880	(0.183–4.226)	0.873			
Dur/Tre treatment line	1st/later	1.375	(0.286–6.603)	0.691			
Data at Dur/Tre introduction							
WBC count	/μL	1.000	(1.000–1.000)	0.619			
Neutrophil count	/μL	1.000	(0.999–1.000)	0.498			
Monocyte count	/μL	1.000	(0.996–1.004)	0.947			
Lymphocyte count	/μL	1.000	(0.999–1.002)	0.916			
NLR		0.782	(0.470–1.299)	0.342			
LMR		1.043	(0.684–1.589)	0.846			
PLR		0.995	(0.982–1.008)	0.421			
Data at 2 weeks after Dur/Tre introduction							
WBC count	/μL	1.000	(1.000–1.000)	0.640			
Neutrophil count	/μL	1.000	(1.000–1.000)	0.409			
Monocyte count	/μL	0.999	(0.995–1.002)	0.451			
Lymphocyte count	/μL	1.001	(1.000–1.003)	0.068			
NLR		0.472	(0.203–1.096)	0.081			
LMR		1.653	(0.965–2.831)	0.067			
PLR		0.973	(0.951–0.995)	0.017	0.981	(0.957–1.004)	0.108
ΔNeutrophil	/μL	1.000	(0.999–1.000)	0.593			
ΔMonocyte	/μL	0.995	(0.988–1.003)	0.203			
ΔLymphocyte	/μL	1.004	(1.001–1.006)	0.016	1.002	(0.999–1.005)	0.130

Abbreviations: HR, hazard ratio; CI, confidence interval; Dur/Tre, durvalumab plus tremelimumab; WBC, white blood cell; NLR, neutrophil-to-lymphocyte ratio; LMR, lymphocyte-to-monocyte ratio; PLR, platelet-to-lymphocyte ratio; Δneutrophil, Δmonocyte and Δlymphocyte; calculated as the change in the amount of neutrophil, monocyte and lymphocyte count between at baseline and at 2 weeks after durvalumab plus tremelimumab introduction.

**Table 3:** Comparison of the radiological best therapeutic response (Response Evaluation Criteria in Solid Tumors version 1.1) to durvalumab plus tremelimumab treatment based on platelet-to-lymphocyte ratio at 2 weeks after durvalumab plus tremelimumab introduction (PLR2w) and the change in the amount of lymphocyte count between at baseline and at 2 weeks after durvalumab plus tremelimumab introduction (Δlymphocyte).

	Whole Cohort	High PLR2w	Low PLR2w	*p* Value	High Δlymphocyte	Low Δlymphocyte	*p* Value
*n*	30	20	10		10	20	
CR	2 (6.7)	0 (0.0)	2 (20.0)		2 (20.0)	0 (0.0)	
PR	7 (23.3)	1 (5.0)	6 (60.0)		5 (50.0)	2 (10.0)	
SD	7 (23.3)	6 (30.0)	1 (10.0)		2 (20.0)	5 (25.0)	
PD	14 (6.7)	13 (65.0)	1 (10.0)		1 (10.0)	13 (65.0)	
ORR	26.7%	5.0%	80.0%	<0.001	70.0%	10.0%	0.002
DCR	53.3%	35.0%	90.0%	0.006	90.0%	35.0%	0.006

Data are presented as *n* (%). Abbreviations: CR, complete response; PR, partial response; SD, stable disease; PD, progressive disease; ORR, objective response rate; DCR, disease control rate; PLR, platelet-to-lymphocyte ratio; PLR2w, platelet-to-lymphocyte ratio at 2 weeks after durvalumab plus tremelimumab introduction; Δlymphocyte, calculated as the change in the amount of lymphocyte count between at baseline and at 2 weeks after durvalumab plus tremelimumab introduction.

**Table 4:** Cox regression analysis to determine the predictors associated with progression-free survival.

		Univariate Analysis			Multivariate Analysis		
		HR	(95% CI)	*p* Value	HR	(95% CI)	*p* Value
Age	years	0.993	(0.949–1.039)	0.758			
Gender	male/female	1.212	(0.407–3.612)	0.730			
Etiology	viral/non-viral	0.987	(0.421–2.317)	0.977			
Dur/Tre treatment line	1st/later	0.832	(0.359–1.926)	0.667			
imAEs	positive/negative	0.239	(0.086–0.667)	0.006	0.321	(0.112–0.923)	0.035
Data at Dur/Tre introduction							
AFP	≥400/<400 ng/mL	1.072	(0.436–2.638)	0.880			
WBC count	/μL	1.000	(1.000–1.000)	0.496			
Neutrophil count	/μL	1.000	(0.999–1.000)	0.399			
Monocyte count	/μL	1.000	(0.998–1.002)	0.933			
Lymphocyte count	/μL	1.000	(0.999–1.001)	0.963			
NLR		1.116	(0.882–1.413)	0.361			
LMR		1.005	(0.783–1.290)	0.969			
PLR		1.002	(0.996–1.007)	0.515			
Data at 2 weeks after Dur/Tre introduction							
WBC count	/μL	1.000	(1.000–1.000)	0.380			
Neutrophil count	/μL	1.000	(1.000–1.000)	0.182			
Monocyte count	/μL	1.000	(0.999–1.001)	0.629			
Lymphocyte count	/μL	0.999	(0.999–1.000)	0.113			
NLR		1.129	(1.003–1.271)	0.044	1.001	(0.854–1.172)	0.994
LMR		0.800	(0.606–1.057)	0.117			
PLR		1.008	(1.001–1.016)	0.023	1.005	(0.995–1.016)	0.317
ΔNeutrophil	/μL	1.000	(1.000–1.000)	0.222			
ΔMonocyte	/μL	1.001	(0.999–1.003)	0.281			
ΔLymphocyte	≥+245/<+245/µL	0.215	(0.072–0.647)	0.006	0.308	(0.095–0.998)	0.049

Abbreviations: HR, hazard ratio; CI, confidence interval; Dur/Tre, durvalumab plus tremelimumab; AFP, alpha-fetoprotein; imAEs, immune-mediated adverse events; WBC, white blood cell; NLR, neutrophil-to-lymphocyte ratio; LMR, lymphocyte-to-monocyte ratio; PLR, platelet-to-lymphocyte ratio; ΔNeutrophil, ΔMonocyte and ΔLymphocyte; calculated as the change in the amount of neutrophil, monocyte and lymphocyte count between at baseline and at 2 weeks after durvalumab plus tremelimumab introduction.

## Data Availability

The data presented in this study are available upon request from the corresponding author.

## Supplementary Materials

The following supporting information can be downloaded at: https://www.mdpi.com/article/10.3390/cancers17081274/s1, Figure S1: Relationships between PLR at 2 weeks after durvalumab plus tremelimumab introduction (PLR2w) and calculated as the change in the amount of lymphocyte count between the baseline and at 2 weeks after durvalumab plus tremelimumab introduction (Δlymphocyte); Figure S2: Kaplan–Meier curves for progression-free survival divided into the plus and the minus of the change in the amount of lymphocyte count between the baseline and at 2 weeks after durvalumab plus tremelimumab introduction (Δlymphocyte); Figure S3: Kaplan–Meier curves for progression-free survival stratified by the optimal value of Δlymphocyte (+245/µL) at 2 weeks after durvalumab plus tremelimumab introduction; Figure S4: Kaplan–Meier curves for overall survival; Table S1: The best radiological therapeutic response (Response Evaluation Criteria in Solid Tumors version 1.1) to durvalumab plus tremelimumab treatment; Table S2: Cox regression analysis to determine the predictors associated with overall survival; Table S3: Immune-mediated adverse events associated with durvalumab plus tremelimumab in the whole cohort; Table S4: Logistic regression analysis to determine the predictors associated with any grade immune-mediated adverse events.

## Abbreviations

The following abbreviations are used in this manuscript:

AFP	alpha-fetoprotein
ALBI	albumin–bilirubin
ALD	alcohol-associated liver disease
Atez/Bev	atezolizumab plus bevacizumab
AUC	area under the curve
BCLC	Barcelona Clinic Liver Cancer
CI	confidence interval
CR	complete response
CT	computed tomography
CTLA-4	cytotoxic T lymphocyte-associated antigen 4
DCP	des-gamma-carboxy prothrombin
DCR	disease control rate
Dur/Tre	durvalumab plus tremelimumab
HCC	hepatocellular carcinoma
HR	hazard ratio
ICI	immune checkpoints inhibitor
imAE	immune-mediated adverse event
IQR	interquartile
LMR	lymphocyte-to-monocyte ratio
LMR2w	LMR at 2 weeks after Dur/Tre induction
MASLD	metabolic dysfunction-associated steatotic liver disease
MRI	magnetic resonance imaging
MTA	molecular targeted agent
NLR	neutrophil-to-lymphocyte ratio
NLR2w	NLR at 2 weeks after Dur/Tre induction
ORR	objective response rate
OS	overall survival
PD	progressive disease
PD-L1	programmed cell death ligand-1
PFS	Progression-free survival
PLR	platelet-to-lymphocyte ratio
PLR2w	PLR at 2 weeks after Dur/Tre induction
PR	partial response
RECIST	Response Evaluation Criteria in Solid Tumors
ROC	receiver operating characteristic
SD	stable disease
STRIDE	Single Tremelimumab Regular Interval Durvalumab
VEGF	vascular endothelial growth factor
WBC	white blood cell
