# Supplementary material for: Usefulness of the Early Increase of Peripheral Blood Lymphocyte Count in Predicting Clinical Outcomes for Patients with Advanced Hepatocellular Carcinoma Treated with Durvalumab Plus Tremelimumab
Source: Cancers (Basel). 2025 Apr 9;17(8):1274. doi: 10.3390/cancers17081274 (PMC12025802; doi:10.3390/cancers17081274)
Supplement: Supplementary file 1 [file cancers-17-01274-s001.zip › Figure S1.pptx]

## Slide 1
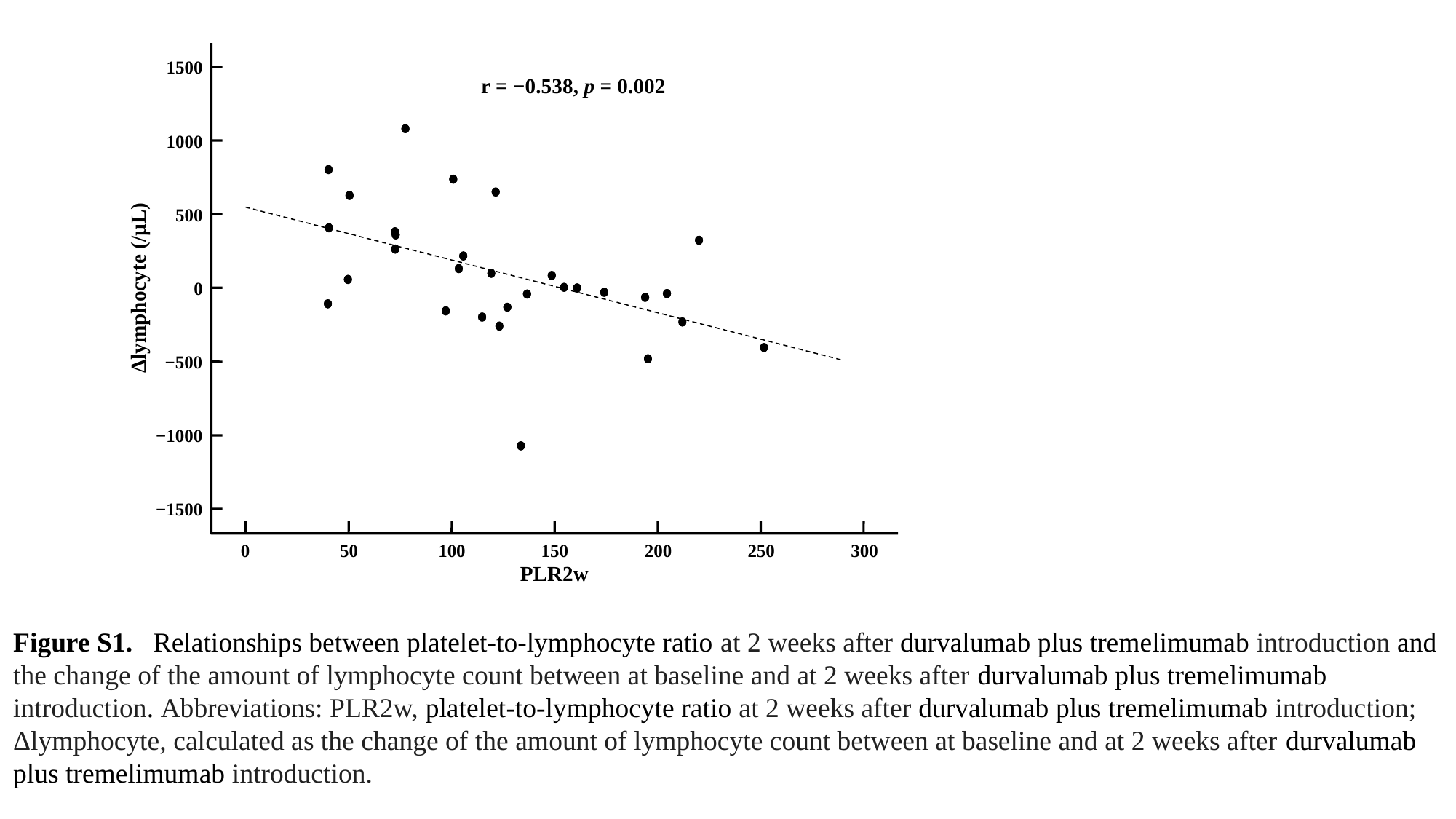

1500
r = −0.538, p = 0.002
1000
500
Δlymphocyte (/µL)
0
−500
−1000
−1500
0
50
100
150
200
250
300
PLR2w
Figure S1. Relationships between platelet-to-lymphocyte ratio at 2 weeks after durvalumab plus tremelimumab introduction and the change of the amount of lymphocyte count between at baseline and at 2 weeks after durvalumab plus tremelimumab introduction. Abbreviations: PLR2w, platelet-to-lymphocyte ratio at 2 weeks after durvalumab plus tremelimumab introduction; Δlymphocyte, calculated as the change of the amount of lymphocyte count between at baseline and at 2 weeks after durvalumab plus tremelimumab introduction.
