# Supplementary material for: Usefulness of the Early Increase of Peripheral Blood Lymphocyte Count in Predicting Clinical Outcomes for Patients with Advanced Hepatocellular Carcinoma Treated with Durvalumab Plus Tremelimumab
Source: Cancers (Basel). 2025 Apr 9;17(8):1274. doi: 10.3390/cancers17081274 (PMC12025802; doi:10.3390/cancers17081274)
Supplement: Supplementary file 1 [file cancers-17-01274-s001.zip › Figure S2.pptx]

## Slide 1
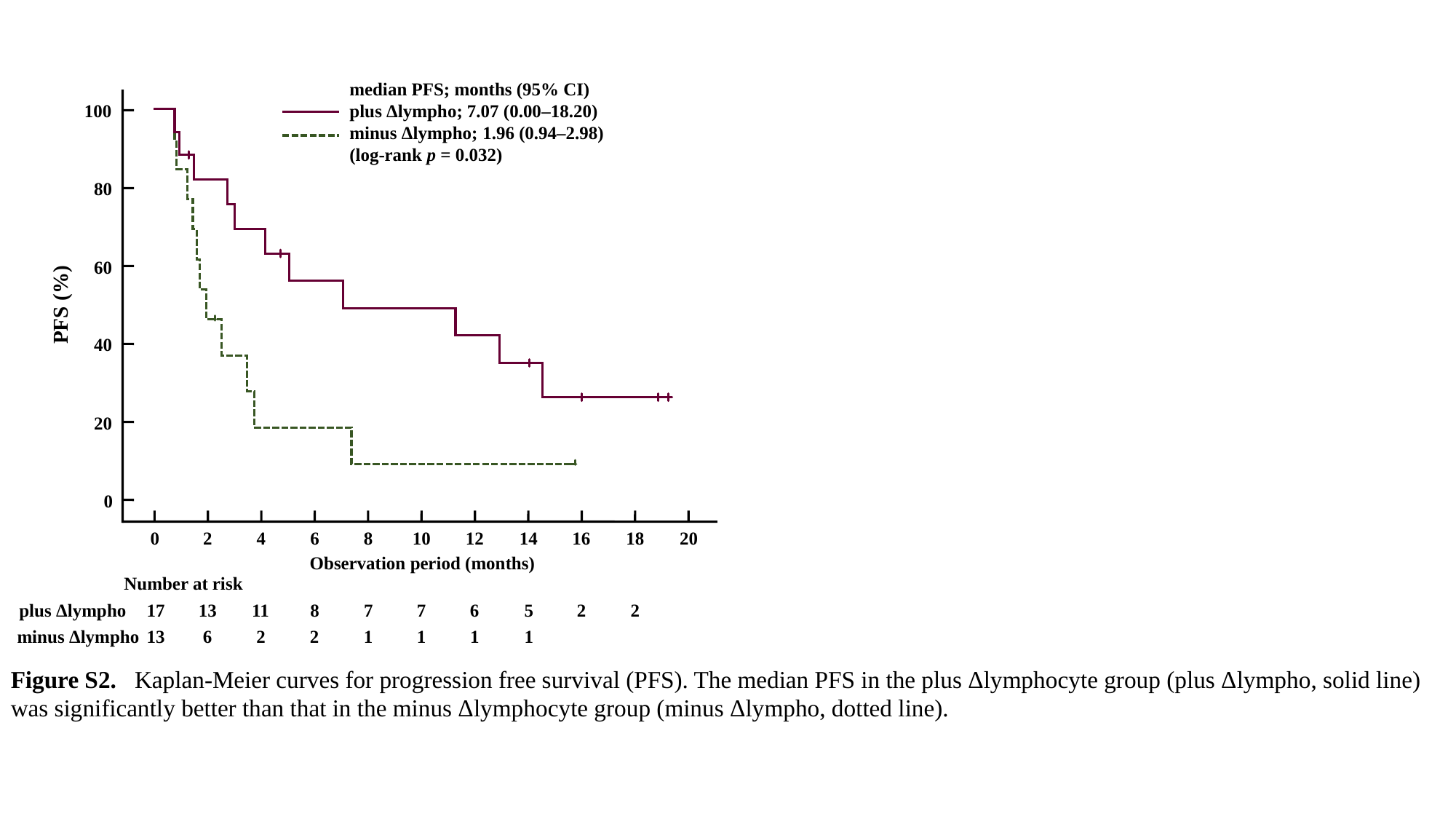

median PFS; months (95% CI)
plus Δlympho; 7.07 (0.00–18.20)
minus Δlympho; 1.96 (0.94–2.98)
(log-rank p = 0.032)
100
80
60
PFS (%)
40
20
0
0
2
4
6
8
10
12
14
16
18
20
Observation period (months)
Number at risk
plus Δlympho
17
13
11
8
7
7
6
5
2
2
1
minus Δlympho
13
6
2
2
1
1
1
Figure S2. Kaplan-Meier curves for progression free survival (PFS). The median PFS in the plus Δlymphocyte group (plus Δlympho, solid line) was significantly better than that in the minus Δlymphocyte group (minus Δlympho, dotted line).
