# Supplementary material for: Usefulness of the Early Increase of Peripheral Blood Lymphocyte Count in Predicting Clinical Outcomes for Patients with Advanced Hepatocellular Carcinoma Treated with Durvalumab Plus Tremelimumab
Source: Cancers (Basel). 2025 Apr 9;17(8):1274. doi: 10.3390/cancers17081274 (PMC12025802; doi:10.3390/cancers17081274)
Supplement: Supplementary file 1 [file cancers-17-01274-s001.zip › Figure S3.pptx]

## Slide 1
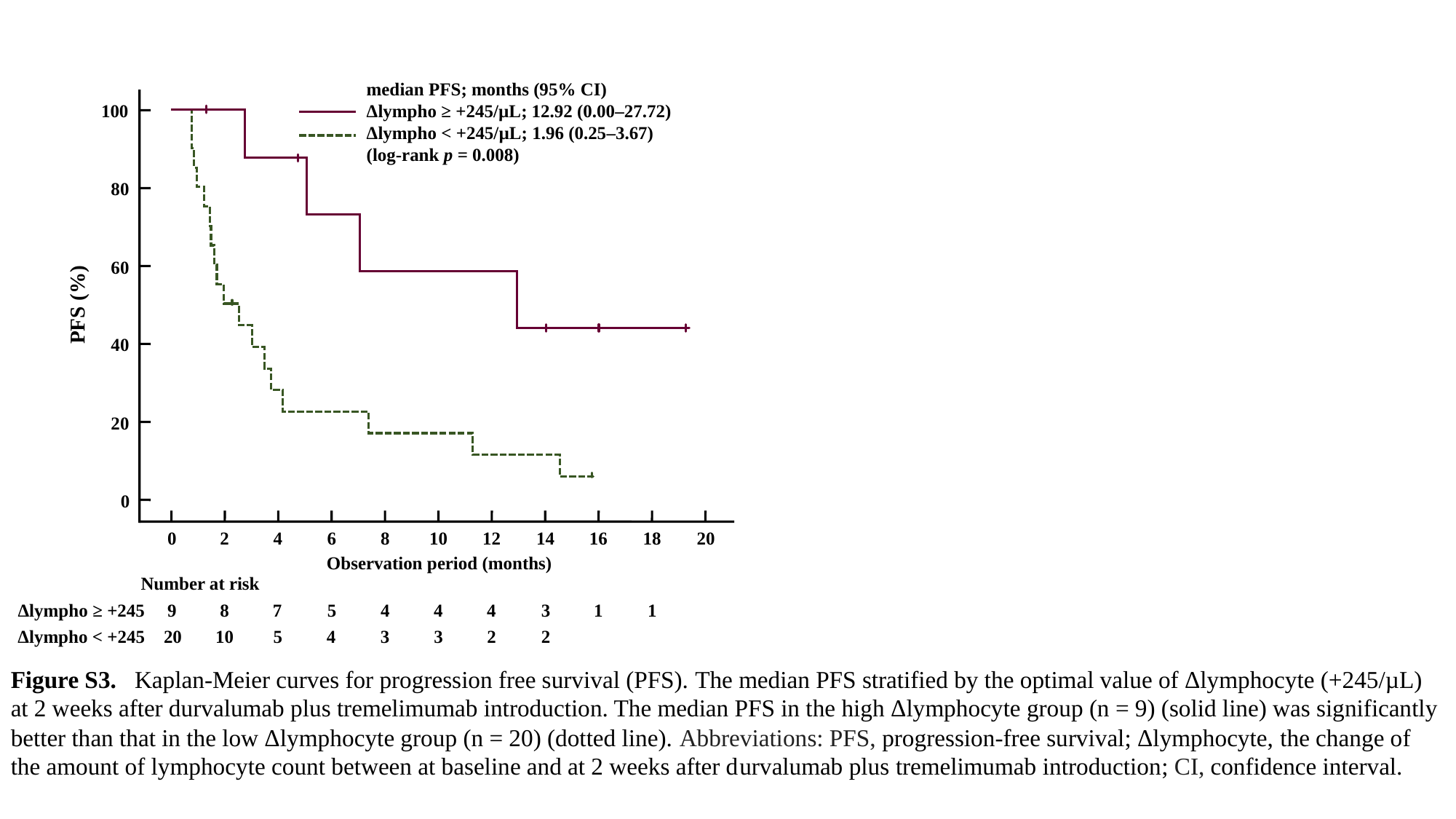

median PFS; months (95% CI)
Δlympho ≥ +245/µL; 12.92 (0.00–27.72)
Δlympho < +245/µL; 1.96 (0.25–3.67)
(log-rank p = 0.008)
100
80
60
PFS (%)
40
20
0
0
2
4
6
8
10
12
14
16
18
20
Observation period (months)
Number at risk
Δlympho ≥ +245
9
8
7
5
4
4
4
3
1
1
2
Δlympho < +245
20
10
5
4
3
3
2
Figure S3. Kaplan-Meier curves for progression free survival (PFS). The median PFS stratified by the optimal value of Δlymphocyte (+245/µL) at 2 weeks after durvalumab plus tremelimumab introduction. The median PFS in the high Δlymphocyte group (n = 9) (solid line) was significantly better than that in the low Δlymphocyte group (n = 20) (dotted line). Abbreviations: PFS, progression‐free survival; Δlymphocyte, the change of the amount of lymphocyte count between at baseline and at 2 weeks after durvalumab plus tremelimumab introduction; CI, confidence interval.
