# Supplementary material for: Usefulness of the Early Increase of Peripheral Blood Lymphocyte Count in Predicting Clinical Outcomes for Patients with Advanced Hepatocellular Carcinoma Treated with Durvalumab Plus Tremelimumab
Source: Cancers (Basel). 2025 Apr 9;17(8):1274. doi: 10.3390/cancers17081274 (PMC12025802; doi:10.3390/cancers17081274)
Supplement: Supplementary file 1 [file cancers-17-01274-s001.zip › Figure S4.pptx]

## Slide 1
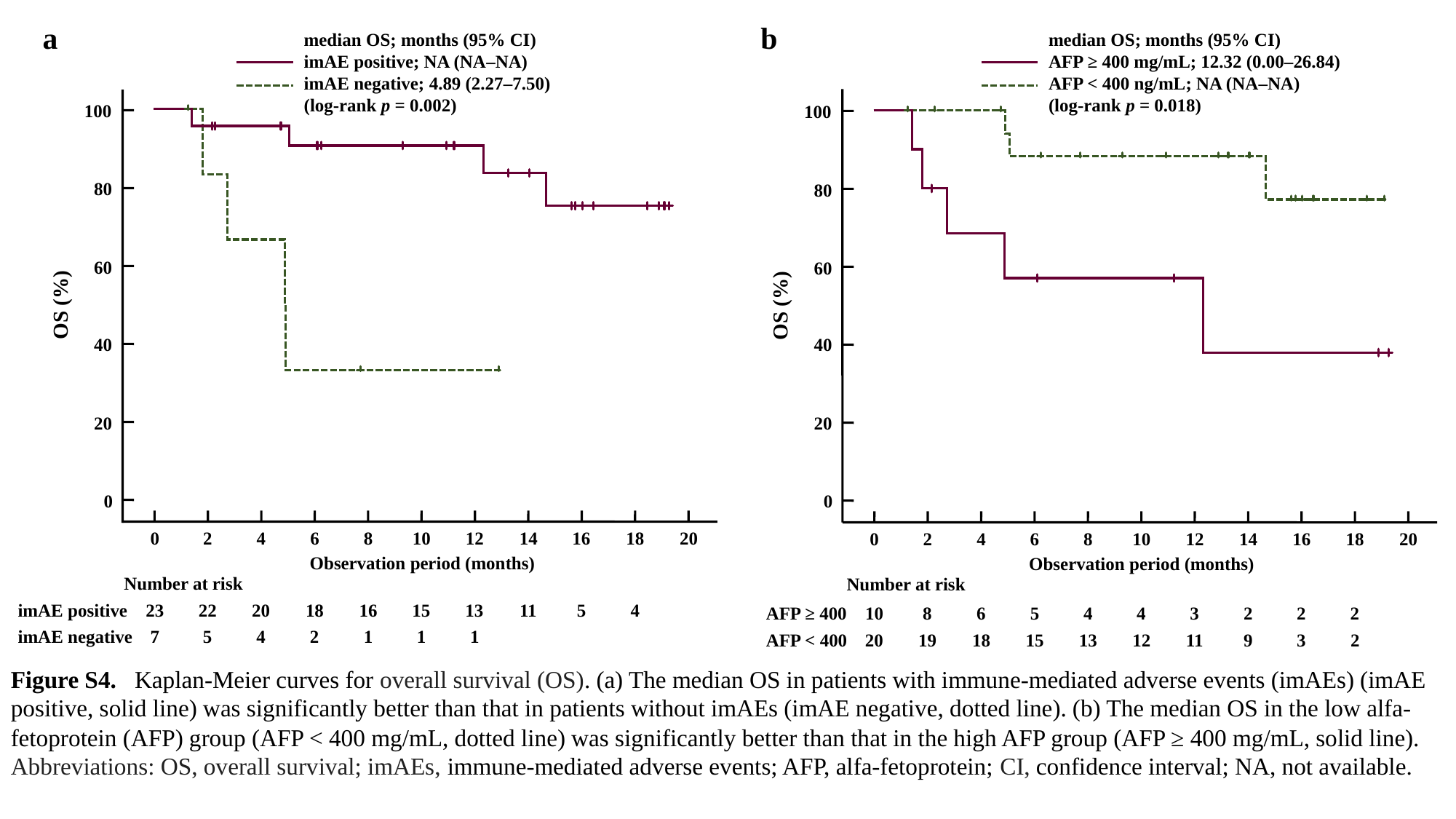

a
median OS; months (95% CI)
imAE positive; NA (NA–NA)
imAE negative; 4.89 (2.27–7.50)
(log-rank p = 0.002)
100
80
60
OS (%)
40
20
0
0
2
4
6
8
10
12
14
16
18
20
Observation period (months)
Number at risk
imAE positive
23
22
20
18
16
15
13
11
5
4
imAE negative
7
5
4
2
1
1
1
b
median OS; months (95% CI)
AFP ≥ 400 mg/mL; 12.32 (0.00–26.84)
AFP < 400 ng/mL; NA (NA–NA)
(log-rank p = 0.018)
100
80
60
OS (%)
40
20
0
0
2
4
6
8
10
12
14
16
18
20
Observation period (months)
Number at risk
AFP ≥ 400
10
8
6
5
4
4
3
2
2
2
AFP < 400
20
19
18
15
13
12
11
9
3
2
Figure S4. Kaplan-Meier curves for overall survival (OS). (a) The median OS in patients with immune-mediated adverse events (imAEs) (imAE positive, solid line) was significantly better than that in patients without imAEs (imAE negative, dotted line). (b) The median OS in the low alfa-fetoprotein (AFP) group (AFP < 400 mg/mL, dotted line) was significantly better than that in the high AFP group (AFP ≥ 400 mg/mL, solid line). Abbreviations: OS, overall survival; imAEs, immune-mediated adverse events; AFP, alfa-fetoprotein; CI, confidence interval; NA, not available.
